# The semantics of Chemical Markup Language (CML) for computational chemistry : CompChem

**DOI:** 10.1186/1758-2946-4-15

**Published:** 2012-08-07

**Authors:** Weerapong Phadungsukanan, Markus Kraft, Joe A Townsend, Peter Murray-Rust

**Affiliations:** 1 Department of Chemical Engineering and Biotechnology, University of Cambridge, New Museums Site, Pembroke Street, Cambridge, CB2 3RA, UK; 2 Department of Chemistry, University of Cambridge, Lensfield Road, Cambridge, CB2 1EW, UK

## Abstract

This paper introduces a subdomain chemistry format for storing computational chemistry data called CompChem. It has been developed based on the design, concepts and methodologies of Chemical Markup Language (CML) by adding computational chemistry semantics on top of the CML Schema. The format allows a wide range of *ab initio* quantum chemistry calculations of individual molecules to be stored. These calculations include, for example, single point energy calculation, molecular geometry optimization, and vibrational frequency analysis. The paper also describes the supporting infrastructure, such as processing software, dictionaries, validation tools and database repositories. In addition, some of the challenges and difficulties in developing common computational chemistry dictionaries are discussed. The uses of CompChem are illustrated by two practical applications.

## Background

### Introduction

Computational Quantum Chemistry is a very popular area of research today and will be even more popular in the future. This is due to several emerging key technologies. Developments in computational quantum theory, better numerical methods, as well as parallel and distributed computing, have significantly reduced the computational time (from months to days or hours). With software packages such as Gaussian [1], GAMESS (US) [2], and GAMESS-UK [3] properties of large or short-lived molecules can be calculated which may be difficult or impossible to obtain experimentally. Increasingly, this is done with little human intervention, as automated chemical model generators are becoming more and more popular [4]. As a consequence the amount of data available will very soon become too vast to be analyzed manually. Regardless of how advanced the technology is, these calculations will always require resources which may be wasted if somebody else has completed the same calculation already. For this reason efficient storage and retrieval of computational chemistry data is an important issue. To address this issue the development of an easily accessible and usable infrastructure is necessary.

At present, most computational results are output as “log files” which are designed to record information as human-readable plain text. The log files contain not only information about the calculated properties, but also metadata, such as computing environments, errors, warnings, etc. Many crucial pieces of information, such as units, computational methods or algorithms, are usually omitted from the outputs because they are often considered to be “obvious” [5] or are provided in separate documentation. Moreover, the structure of the log files depends on the software used, which creates difficulties in retrieving textual information among the different formats. This impedes the automation of the data analysis which is essential in the study of a large chemical system.

A typical solution to the problem is to extract the information from the log files (known as “parsing”) and cast them into a format that is more efficient for retrieval and processing. The eXtensible Markup Language [6] (XML) is usually selected for storing data due to its universality and extensibility for both simple and complex data. Furthermore, XML provides the means for checking conformance of the structure and data ensuring that the XML instances meet the requirements of the application in question. The fact that XML has become an industrial standard for data storage, in addition to the fact that most modern software is built to support it, are the strongest testaments to its usefulness.

For chemistry applications, the Chemical Markup Language (CML) [7-10] has been developed based on the XML standard in order to provide the semantics for chemical data. CML allows the representation of complex chemical objects by using the hierarchical tree structure of XML. In addition, CML is accompanied by a number of methodologies [11-13] and infrastructures, such as CMLXOM [14], Jumbo6 [15], Jumbo-Converter [16] and CMLValidator [17], which support the development of a more general computational chemistry format. The following features make CML specifically suited for our purpose: 

1. CML contains a set of hundreds of chemical name tags covering all aspects of chemistry and so allows one to compose a suitable representation for any chemical data;

2. CML is widely supported by chemistry software, such as, OpenBabel [18], PyBel [19], Jmol [20], Avogadro [21], making it easy to integrate a subdomain format of CML into most of the existing systems which use these libraries with little modification;

3. CML has been developed over 15 years so the terminology, concepts and semantics have become highly stable, complete and well understood with relatively small changes in its schema and, as a result, it has been accepted by the chemistry community.

The **purpose of this paper** is to use CML to develop a standard called CompChem, which is suitable to represent computational chemistry information, including a set of supporting open-source tools. Furthermore, we illustrate the use of CompChem for managing computational chemistry data and for calculating thermodynamic properties.

The paper is structured as follows. We briefly review the important CML concepts used throughout this paper in section “CML overview”. In section “Methodology in CompChem”, we describe the requirements for the design of CompChem and the semantics and the detailed specification of CompChem. Finally, in section “Utility : example use cases”, we report a recent application with examples.

### CML overview

In this section, we briefly outline the key CML concepts and terminologies, which are adopted by CompChem, for readers who are not familiar with CML. Detailed discussions have already been published in *Murray-Rust et al.*[13] and *Townsend et al.*[11]. The latest information of the ongoing developments are also publicly available online at http://www.xml-cml.org. The development of CompChem is based on the following components and concepts: 

· XML Schema [22] is an XML-based schema language which specifies the constraints on the structure of an XML document. It is also written in XML and referred to as XML Schema Definition (XSD). The term “XML Schema” (with a capital “S”) should not be confused with XML schema. The latter is a term describing schema languages in general. XML Schema is one of the most commonly used schema languages today. It was published as a W3C recommendation in 2001 [23] to replace Document Type Definition (DTD) and provide additional features for defining the constraints and validating the contents of XML document.

· CML Schema [10, 24] is an XML Schema containing hundreds of chemical definitions (XML tags and attributes). It covers most aspects of chemistry, e.g., CMLReact [25] for chemical reactions, CMLSpec [26] for spectral data, CML for crystallography [27] and CML for polymers (PML) [28]. With the CML Schema, one can determine if a CML document conforms to the specification or not. For example, the schema will tell whether a CML document contains a misspelled element name or an undefined attribute. This ensures that the applications will not generate any errors due to using a “bad” CML document as their input. In the latest version of CML Schema (version 3), the content model restrictions have been lifted in order to make it more flexible for creating any type of chemical documents.

· CML Convention is a set of rules and constraints on the content model of a CML document. It is a subset of the CML Schema with some additional rules for a specific chemistry domain, some of which cannot be defined using XSD. When a convention is specified on a CML element (using the @convention attribute), the structure of the element must conform to the rules defined by the convention. The convention is represented by a short-hand notation, known as a qualify name (QName [29]), which represents a globally unique Uniform Resource Locator (URL).

· CML Dictionary is a collection of “controlled vocabularies” which are used to add semantics to generic CML elements, especially for <parameter> and <property>. There are several types of CML dictionaries, for example, property and parameter dictionaries (specified using @dictRef), unit dictionaries (specified using @unit) and unit type dictionaries (specified using @unitType). The existing dictionaries can be found at http://www.xml-cml.org/dictionary/.

**Figure 1:**
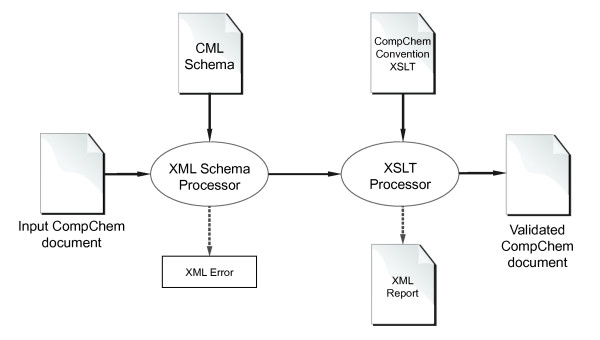
A linear schematic diagram of validation process for CompChem.

· Validation is the most important step to verify whether a CML document conforms to the structure required by your application. The CML approach to validation [11] consists of several steps, e.g., CML Schema, CML convention, CML dictionary validations, and so on. These are usually performed sequentially (as shown in Figure 1), however, they are completely independent. A sophisticated online validator is available at http://validator.xml-cml.org/.

## Methodology in CompChem

### CompChem design

The development of CompChem started back in the summer of 2009 with the initial goal of archiving our published computational quantum chemistry results [30-34], which were calculated using the convenient software Gaussian 03, in a machine readable format and stored in a queriable database for automating the studies of chemical reactions in a combustion system. It was a collaborative effort between chemical engineers and cheminformatic scientists to explore the power of Semantic Web technologies for storing scientific data. The format was developed purely using the existing CML without making any modification to its schema. The number of elements we use in CompChem, see sections “CompChem convention” and 2, is currently relatively small compared to the whole set of CML elements available, but it is sufficient for most of the data that needs to be stored in the current work. It is very likely that other CML elements will be included to support other functionalities in later years as CompChem evolves.

Like other XML standards, the CompChem convention can only work well if it is widely accepted and, until now, there has not been one for computational chemistry, due to the varied nature of studies. This is a fact that we have to accept and, therefore, we only focus on formalizing the data calculated from the quantum chemistry software in this work.

The design of the CompChem convention shares and inherits the common goals of CML, Polymer Markup Language (PML) and other XML standards, which are quoted from XML 1.0 W3C Recommendation [6]. (Readers are advised to read this documentation for further details) These are as follows: 

1. CompChem shall be straightforwardly usable over the Internet;

2. CompChem shall support a wide variety of applications;

3. CompChem shall be compatible with Standard Generalized Markup Language (SGML);

4. It shall be easy to write programs which process CompChem documents;

5. The number of optional features in CompChem is to be kept to the absolute minimum, ideally zero;

6. CompChem documents should be human-legible and reasonably clear;

7. The CompChem design should be prepared quickly;

8. The design of CompChem shall be formal and concise;

9. CompChem documents shall be easy to create;

10. Terseness in CompChem markup is of minimal importance.

Apart from these general goals, there are more specific goals which distinguish CompChem from CML and other XML standards: 

1. CompChem should be based on CML and reuse its components where appropriate. This is a typical goal of all subdomain formats of CML. Reusing CML and its components is the fundamental key to improve the quality and consistency of the format and reduce development cost and effort. In addition, any future improvement made into CML and its technologies will also be immediately applied to CompChem. In the development of CompChem, we introduced no new components into the CML Schema. Instead, the new concepts are defined using CML dictionaries and are applied to generic CML containers, see Section “Using dictionary in CompChem”.

2. CompChem should capture the semantics of most computational chemistry calculations. This is the main goal of our work. It is to reduce the flexibility in CML Schema and introduce a stricter structure into the documents so that software and applications know exactly how to process the information. The semantics of CompChem is modelled based on the typical nature of computational simulations or calculations, i.e., contains model input and output steps, see Section “CompChem convention”.

3. CompChem shall support any chemical data. CML provides a rich set of chemical data types in addition to standard XML data types. It is also possible to build more complex chemical objects from the abstract CML data types and components, thus, CompChem has gained this advantages from reusing CML.

4. CompChem should be able to be validated using standard processing tools. This is an important consideration to make the CompChem platform independent. The development of CompChem involves using both CML components and CML technologies. The CML components, i.e., CML elements and attributes, are validated using CML Schema and any standard XML Schema processor. The XML stylesheet, XPath [35] and XSLT [36] are chosen for implementing and validating the CML conventions. Therefore, one should be able to validate the CompChem convention by using any web browser capable of rendering XSLT.

5. CompChem should represent both computational input and output. CompChem is designed to be used as both input and output for the calculations. The computation input contains critical information, such as calculation model, basis set, level of theory, job type, etc., that defines the calculation itself. This information is required for the search functionality of the digital repository and the calculation output is usually what is returned from the search. Being able to store input and output are required features of CompChem.

6. CompChem should interoperate with other XML or CML models (conventions). This is one of the common goals that is shared by all CML works. Interoperability is a requirement for CompChem to be used in conjunction with other existing XML-based formats such as Dublin CoreⒸMetadata (DCMI) and Object Reuse and Exchange (OAI-ORE) standards. This makes CompChem not only reuse the CML components but also other well established formats.

7. CompChem shall allow users to define and insert new concepts. As discussed earlier, new concepts are added into CompChem through the use of a dictionary mechanism. This is not only applied to the basic values, such as <property>, <parameter>, @unit and @unitType, but also the complex model objects. It is feasible to insert an entire new convention into CompChem, although, it may not be understood by all standard chemistry tools.

8. CompChem Convention rules must be clear and well documented. Although the convention rules are implemented into the CompChem convention validator using stylesheets, it is important that there must also be human readable documentation. Clear documentation benefits both users and developers in the long term. We will adhere to this in all of our development. In practice, we make the decisions on what are the rules that should be in CompChem and then write documentation from these rules. After that, we implement the rules into the convention validator. This discipline ensures that there is always documentation for every convention we develop.

### Using dictionary in CompChem

Because dictionaries play a central role in defining the semantics within a CompChem document, it is essential to fully understand the concepts and how the dictionary referencing mechanism works. Both are explained in detail in this section.

Concepts are the building blocks of scientific knowledge. In natural language, similar concepts can be expressed using several words or synonyms which are the common causes of ambiguity, confusion and error when the information is being processed. In software development, several similar concepts or synonyms can be grouped and represented by a carefully pre-determined term or vocabulary, commonly known as *controlled vocabulary*. Using controlled vocabulary, one can impose an order and reduce ambiguity by allowing the same concepts to be labelled using a single unique term.

In XML, the tags and attributes are predetermined terms, in other words, an XML schema is a set of controlled vocabularies. CML is no exception. The CML elements and attributes are predefined to cover almost all general aspects of chemistry and computational chemistry. However, it is impossible and futile to predefine every possible chemistry concept into CML. For example, concepts like boiling point, melting point, basis set, entropy, enthalpy, methodology, algorithm, etc., are not included in the CML Schema. Instead, CML uses a dictionary and a referencing mechanism to specify a new concept on the generic CML containers, such as <parameter>, <property>, <scalar>, <matrix>, etc., which can be used to hold the values of any types.

A new concept can be added as an entry into a CML dictionary without requiring the CML Schema to be modified. The dictionary referencing mechanism consists of 3 steps; **defining the new concept**, **creating a reference** to the defined concept and **applying the reference** to the CML generic container. 

**Figure 2:**
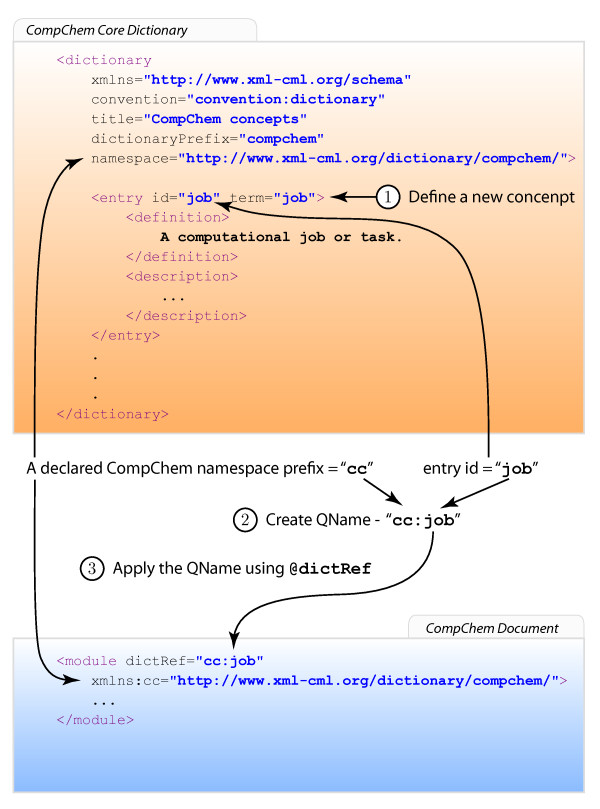
**Diagram illustrating the dictionary referencing mechanism using @dictRef in 3 steps.** A snippet of the dictionary and its entry are shown in the top (orange) box and a snippet of CompChem job module is show in the bottom (blue) box.

· Defining a new concept. In Figure 2 (1), we show a snippet of a CML dictionary which is created according to the CML dictionary convention. A dictionary can contain multiple child elements of entries allowing the vocabulary in the same category to be grouped as one set. The figure only briefly illustrates how a dictionary and its vocabulary should be defined so readers are strongly advised to read the latest detailed specifications of the dictionary convention on www.xml-cml.orgfor more information.

· Creating a reference to the defined concept. In CML, a qualify name (QName) [29] is used to identify an entry in the dictionary. A QName contains a namespace URI [29], a local part and a prefix. The prefix is only used as a placeholder for the associated namespace URI and is declared in a namespace declaration. Therefore, in order to be able to identify the dictionary, each dictionary must have a unique identifier and it is specified using @namespace on <dictionary>. This is not to be confused with the XML namespace which is denoted by @xmlns. Specifying @namespace on <dictionary> does not change the actual XML namespace of <dictionary>; it remains in the CML namespace (http://www.xml-cml.org/schema).Each entry must have a unique @id (unique within the dictionary) and this is used as the local part of the QName. The combination of the dictionary @namespace and entry @id generates a globally unique reference for the defined concept. In Figure 2 (2), the prefix “cc” is associated to the same URI (http://www.xml-cml.org/dictionary/compchem/) that is declared for the CompChem-core dictionary’s @namespace. Using the entry id “job”, a QName “cc:job” is constructed as a reference in this step.

· Applying the reference. The reference or QName can be applied to a container using @dictRef, shown in Figure 2 (3).

This referencing mechanism is not only applied to @dictRef but also @units, @unitType and other attributes. Although the mechanisms are similar, the unit and unit type dictionaries are not defined using <dictionary> but rather <unitList> and <unitTypeList> respectively. This is because the unit and unit type are common concepts for scientific data so it has been defined in the CML Schema.

### CompChem convention

According to our design criteria that CompChem convention should capture the typical underlying processes of quantum calculations and their relationships, the proposed architecture described here is broad and may be applied to any computational modeling in general. The core concepts of CompChem contain the following components:

1. Job list(jobList) In computational quantum chemistry, calculations are often comprised of a series of subtasks, e.g., coarse optimization → fine optimization → NMR Spectrum Analysis. Each job performs a different type of calculation and passes the results to the next calculation job; this is because most quantum chemistry software packages are designed to be modularized and only to perform a single task at a time. The jobList concept is introduced to capture this series of successive subtasks and links the information from one subtask to the next subtask. It behaves like a wrapper for job modules.

2. Job(job) The job concept represents a computational job or a computer simulation task, e.g., geometry optimization and frequency analysis jobs, performed by quantum chemistry software. The job concept is the smallest module that fully describes an overall picture of a computational modeling unit. It consists of model parameters (initialization) and model optimizations or calculations (calculation), model results (finalization) and computing environments (environment). These four components are fundamental to every simulation. However, it is not required that all four components be present in every job. Only model parameters are mandatory. A module that contains only model parameters may be used as an abstract quantum chemistry input.

3. Model initialization(initialization) The model initialization concept represents the model parameters and inputs for a computational job. The model parameters are one of the most important elements that exist in every modeling study. Therefore, it is required in the CompChem convention.

4. Model calculation(calculation) A model calculation concept represents the computation, the optimization or the iteration processes for the computational job specified by the initialization. The calculation process may or may not be of interest to some scientists; therefore, it is an optional information in CompChem.

5. Model finalization(finalization) A model finalization concept represents the model output or result of a computational job. In some cases, a CompChem module may only represent the model inputs and does not contain any calculations, therefore, it is optional in CompChem.

6. Computing environment(environment) The computing environment concept refers to the configuration settings with respect to the hardware platform, software application and operating system. The environment also includes metadata such as machine id, username, starting and finishing date time, tools, compilers, and Internet Protocol address (IP address).

7. User defined concept CompChem allows users to define their own concepts if the recommended concepts above do not fit into their requirements. A user defined concept in CompChem is represented by a module element with a @dictRef attribute whose value points to an entry in a dictionary that defines the concept. Users are free to design any structure for a user defined module. However, it is recommended to use existing structures or a structure that has a schema for validation. Information in a user defined module cannot be guaranteed to be understandable by all processing software tools.

Each concept, defined above, is associated with the core CompChem dictionary (available at http://www.xml-cml.org/dictionary/compchem/), whose @dictRefs and rules are given in Table 1. The rules in this table are coded into a stylesheet which can be used to validate a CompChem document. It is anticipated that the rules need to be modified or extended when more complex calculations, such as transition state searches or molecular dynamic simulations are included in CompChem.

**Table 1:** Rules of CompChem

**dictRef.**	**Rules**
cc:jobList	- A jobList module element MUST have an id attribute the value of which MUST be unique within the module specifying the compchem convention.
	- A jobList module element MUST contain at least one job module child element.
	- A jobList module element SHOULD have a title attribute the value of which MUST be a non-empty string specifying a human-readable title for the module.
	- A jobList module element MAY contain more than one child element in any namespace.
cc:job	- A job module element MUST contain exactly one initialization module child element.
	- A job module element MAY contain zero or more calculation module child elements.
	- A job module element MAY contain no more than one finalization module child element.
	- A job module element MAY contain no more than one environment module element.
	- The order of the calculation module elements in a job module MUST represent the order of the calculation steps but there is no restriction on the order of other child element types.
	- If a calculation module element is present, a finalization module element MUST also be present as a child of a job module element.
	- A job module element SHOULD have a title attribute, the value of which MUST be a non-empty string specifying a human-readable title for the module.
	- A job module element MAY also contain other child elements in any namespace.
cc:initialization	- An initialization module element MUST NOT contain more than one <molecule> child element. The <molecule> MUST specify a convention using the convention attribute and the convention SHOULD be one of the RECOMMENDED molecular conventions.
	- An initialization module element MUST NOT contain more than one <parameterList> element.
	- An initialization module element MAY contain any number of user defined module element.
	- An initialization module element MUST contain at least one child of molecule, <parameterList> or user defined module elements.
	- An initialization module element MAY contain more than one child element in any namespace but MUST NOT contain a property child element or a <propertyList> child element.
	- A job module element MAY also contain other child elements in any namespace.
cc:initialization	- An initialization module element MUST NOT contain more than one <molecule> child element. The <molecule> MUST specify a convention using the convention attribute and the convention SHOULD be one of the RECOMMENDED molecular conventions.
	- An initialization module element MUST NOT contain more than one <parameterList> element.
	- An initialization module element MAY contain any number of user defined module element.
	- An initialization module element MUST contain at least one child of molecule, <parameterList> or user defined module elements.
	- An initialization module element MAY contain more than one child element in any namespace but MUST NOT contain a property child element or a <propertyList> child element.
cc:calculation	- A calculation module element MUST NOT contain more than one molecule child element. The molecule MUST specify a convention using the convention attribute and the convention SHOULD be one of the RECOMMENDED molecular conventions.
	- A calculation module element MUST NOT contain more than one <parameterList> element.
	- A calculation module element MUST NOT contain more than one <propertyList> element.
	- A calculation module element MAY contain any number of user defined module elements.
	- A calculation module element MUST contain at least one child of molecule, <parameterList>, <propertyList> or user defined module elements.
	- A calculation module element MAY contain more than one child element in any namespace.
cc:finalization	- A finalization module element MUST NOT contain more than one molecule child element. The molecule MUST specify a convention using the convention attribute and the convention SHOULD be one of the RECOMMENDED molecular conventions.
	- A finalization module element MUST NOT contain more than one <propertyList> element.
	- A finalization module element MAY contain any number of user defined module elements.
	- A finalization module element MUST contain at least one molecule child, <propertyList> child or user defined module element.
	- A finalization module element MAY contain more than one child element in any namespace but MUST NOT contain a parameter child element or a <parameterList> child element.
cc:environment	- An environment module element MUST NOT contain more than one <propertyList> element.
	- Any environment property element MUST be a child of a <propertyList> element.
	- An environment module element MAY contain more than one child element in any namespace including any number of user defined module elements. However, CompChem can only understand a particular set of concepts.
	- An environment module MUST contain at least one child of <parameterList> or userDefinedModule elements.
	- An environment module element MAY contain more than one child element in any namespace but MUST NOT contain a parameter child element or a <parameterList> child element.

The keywords “MUST”, “MUST NOT”, “REQUIRED”, “SHALL”, “SHALL NOT”, “SHOULD”, “SHOULD NOT”, “RECOMMENDED”, “MAY”, and “OPTIONAL” in this document are to be interpreted as described in RFC 2119 [37].

**Figure 3:**
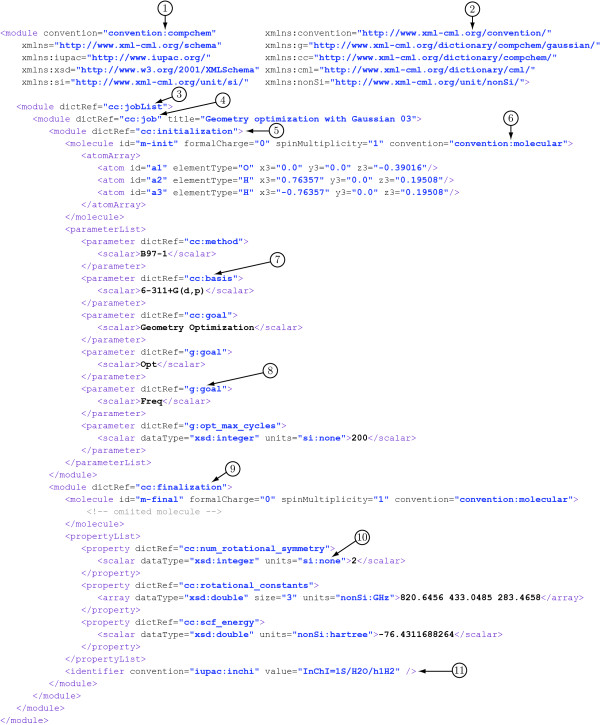
The structure of CML for storing computational chemistry output: (1) CompChem convention declaration, (2) CML convention namespace, (3) a jobList module, (4) a job module, (5) an initialization module, (6) Molecular convention declaration, (7) a basis set parameter specified by cc:basis dictionary reference, (8) a Gaussian specific parameter declared in Gaussian dictionary, (9) a finalization module, (10) si:none for dimensionless units, (11) CML identifier.

Figure 3 shows a snippet of a CompChem document with the key features labeled accordingly.

### Semantics of properties and parameters

There is a core set of CML which is required for storing the actual contents and data. Since CML Schema are content model free, it is necessary to precisely define how the elements should be used. In this section, we list and describe the CML elements which are often found to be useful in CompChem documents. The rules given here for these components are meant to serve only as a guideline for using the common CML components, such as <property>, <parameter>, <scalar>, <array>, and <matrix>. If the given rules are not applicable, users are allowed to define their own structures and annotate it with their own dictionary reference using the @dictRef attribute. However, the new structures should be clearly specified and documented in the user dictionary so that anyone is able to write a code that can process the dictionary.

#### Parameter and property containers

A container is a general notion for an XML element that contains data. The CompChem element parameter is also a container. The exact definition of parameter depends on the context where it is used. In the context of CompChem, parameters are a set of model conditions which can be numerical quantities, options, constraints, text or any chemical objects, for example, a basis set (e.g., 6-311+G(d,p)), level of theory, convergence criteria, calculation type (e.g., geometry optimization, frequency analysis, NMR). Some values can be enumerated. For example, Gaussian 03/09 [1] may need to know whether it should use symmetry in the wave function or not. This option can be set to only either “NoSymm” or “Symm” according to the online manual for Gaussian software [1] and this can be pre-enumerated for use in a CompChem document with values “On” or “Off”.

**Figure 4:**
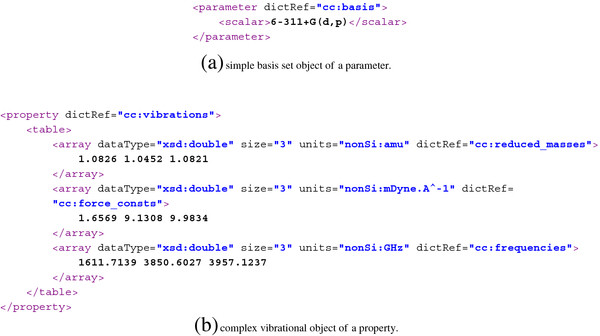
Simple (a) and complex (b) objects in CML.

In CompChem, a value cannot be added directly as a text child of a parameter. It must be wrapped by a CML primitive data container, see Section “Data containers”, which is usually one of <scalar>, <array> or <matrix>. For plain text, a scalar should be used. This allows the computer software to understand exactly which variable type (i.e., variable type in programming language) is suitable for the value of a given parameter. In many cases, a primitive container is not sufficient and it requires a complex object representation to hold the data. Figure 4 shows examples of both primitive and complex chemistry objects. In Figure 4(b), we illustrate a complex object using <table>.

Similar to parameter, a property is also another CML generic container which is used to wrap any primitive or complex object data type. In the context of CompChem, properties are derived quantities from the output of the model calculation, for example, a set of vibrational frequencies of a molecule, electronic energy, derived thermodynamical properties from statistical mechanics calculations. It is often found that properties are numerical quantities rather than enumerated values or text so primitive containers such as <scalar>, <array> and <matrix>, are usually sufficient for storage. For complex objects, they are supported in exactly the same ways as for the parameters.

CompChem also uses @dictRef to provide the semantics for parameter and property. For example, in Figure 3, a parameter has a @dictRef value of cc:basis which points to a cc:basis entry in a CompChem dictionary. Thus, this parameter can be interpreted using the definition of the associated dictionary entry, i.e., cc:basis.

However, there is one exception for molecule elements. Although, an initial molecular geometry can be considered as a model parameter or a model input, CompChem does not categorize it as parameter or property. This is to avoid creating unnecessary concepts and to distinguish the molecule, which is fundamental to every computational chemistry calculation, from other parameters and properties. The semantics of a molecule is considered to be implicit and is determined by its location in the CompChem document. For example, if a molecule is a child of initialization or calculation module, it is considered as an input, i.e., parameter, of that model or calculation. If it is found as a child of finalization module, it is considered to be an output, i.e., property, of the model.

#### Data containers

CML provides elements to hold many different types of mathematical, scientific and computational values, e.g., scalar, vector, matrix, array, etc., which we will refer to as “data containers”. The rules of the key containers are given in Table 2. We will briefly describe the more commonly used data containers. 

· scalar is used to hold scalar data, which is a single value of type integer, real, boolean, string, date, etc.

· array is used to hold a one dimensional array data structure of primitive data type such as integer, real or boolean but it is not suitable for all data types such as string and date, for example.

· matrix is used to hold a two-dimensional rectangular matrix data structure of primitive data type such as integer and real, and it is not suitable for all data types such as string, date or boolean, for example.

· zMatrix In many quantum chemistry calculations, some atomic coordinates are represented using a z-Matrix coordinate system. CompChem adopts the <zMatrix> from the CML schema and uses it as container for <length>, <angle> and <torsion>.

**Table 2:** Rules of data containers

**CML element**	**Rules**
<scalar>	- A <scalar> MUST conform to the CML Schema.
	- The data type of <scalar> is REQUIRED and MUST be specified using a @dataType attribute. The value of @dataType attribute MUST be a primitive data type, e.g., xsd:integer, xsd:double, xsd:real, xsd:float, xsd:boolean, etc.
	- A <scalar> MUST have units unless the @dataType is an xsd:string. (si:none for dimensionless units).
	- A <scalar> MUST NOT have unit and unit type if the @dataType is an xsd:string.
<array>	- An <array> MUST conform to the CML Schema.
	- The data type of <array> is REQUIRED and MUST be specified using the @dataType attribute. The value of @dataType attribute MUST be a primitive data type, e.g., xsd:integer, xsd:double, xsd:real, xsd:float, xsd:boolean, etc., but it MUST not be an xsd:string.
	- An <array> MUST have units even if they are dimensionless (si:none for dimensionless units).
	- The size of <array> is OPTIONAL and is specified using the @size attribute with the minimum value of 1.
	- The @delimiter attribute is OPTIONAL. If not set, the array entries are separated by whitespace.
<matrix>	- A <matrix> MUST conform to the CML Schema.
	- The data type of <matrix> is REQUIRED and MUST be specified using the @dataType attribute. The value of @dataType attribute MUST be a primitive data type, e.g., xsd:integer, xsd:double, xsd:real, xsd:float, xsd:boolean, etc., but it MUST not be an xsd:string.
	- A <matrix> MUST have units even if it is dimensionless (si:none for dimensionless units).
	- The dimension of a <matrix> is REQUIRED and MUST be specified using @rows and @columns attributes with the minimum values of 1.
	- The @delimiter attribute is OPTIONAL. If not set, the matrix entries are separated by whitespace.
<zMatrix>	- A <zMatrix> MUST conform to the schema of CML matrix.
	- A <zMatrix> SHOULD be a child of a <molecule> in molecular convention.
	- A <zMatrix> MAY contain any number of <length>, <angle> and <torsion>, which MUST also conform to the CML Schema.

The keywords “MUST”, “MUST NOT”, “REQUIRED”, “SHALL”, “SHALL NOT”, “SHOULD”, “SHOULD NOT”, “RECOMMENDED”, “MAY”, and “OPTIONAL” in this document are to be interpreted as described in RFC 2119 [37].

## Utility : example use cases

### MolHub

**Figure 5:**
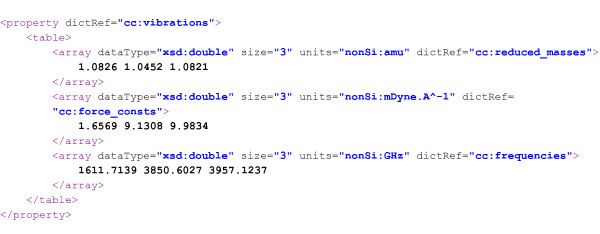
MolHub - data repository for computational quantum chemistry.

MolHub is an online infrastructure for chemical data that is used in combustion kinetic studies (http://como.cheng.cam.ac.uk/molhub/), a web browser snapshot is shown in Figure 5. Its architecture is highly flexible allowing add-on modules, i.e., plugins, to be added independently. It was originally named “CoMo CompChem” (CMCC), which was published as part of *Shirley et al.*[33] for determining thermochemistries and studying the equilibrium of new titanium gas phase species which are involved in an industrial rutile chlorinator.

In MolHub the operating data resources are mainly in XML format (CompChem for computational chemistry data) but it also offers alternative access to the raw data (in legacy format), in the case that the XML formats do not contain the required information. The resources are uniquely identified by URLs and linked semantically by the Resource Description Framework (RDF) [38] allowing the data to be accessed and queried using standard HTTP protocol. The design of URLs and services are based on the REpresentational State Transfer (REST) principles in which the URL represents the location of the resource and the HTTP method represents the operation that can be applied to the resources.

The MolHub online service can be accessed either directly from a web browser or from within software. Since MolHub’s core API is based on the pure HTTP protocol, it is possible to use almost any programming language that provides HTTP libraries, e.g., httplib in Python, URLConnection and HttpClient in Java, libcurl in C++, etc. We achieve the goal of creating a collaborative environment, while at the same time allowing the use of the programming language that works best in the developer’s environment. However, simple web interfaces such as a form to upload data are also provided. Users can access these features through the web browser without additional tools, allowing them to easily interact with MolHub. The web frontend is built using standard HTML5 and Javascript, in which the Javascript codes communicate with our core API using Ajax (Asynchronous JavaScript and XML).

### Example A: Indexing computational chemistry data

Semantics in CompChem are implicit, i.e., the relationships of elements are conveyed based on a mutual understanding (not by RDF [38] and OWL ontologies [39]). The implicit semantics of CompChem can be easily translated into RDF allowing each resource to be identified and related in the form of subject-predicate-object triples (RDF statements). So far, there exist no ontology for computational chemistry which can be used as a starting point for a semantic conversion from CompChem to RDF. The development of relationships in RDF is currently based on the demand for very specific applications. The graph database (Triple store for RDF) has proven to be easy to understand and maintain (in comparison to multiple tables in a relational database management system), especially for scientific data in which the information is not frequently changing all the time.

At the current stage, MolHub has been developed to support the data of Gaussian 03 calculations (by converting into CompChem format) providing several online services for calculating thermochemistries of existing online molecular resources. It automatically converts the uploaded Gaussian log files into CompChem, RDF, HTML, N3 (Notation3, an RDF alternative) and PNG (Portable Network Graphics) images. The RDF files are added to a triple store, i.e., we use OpenRDF [40] in this work, offering a queriable back-end through SPARQL [41]. Various data formats are viewable from the web browser without any additional software which makes it easy for users to explore, and for search engines to discover and index our data.

### Example B: titanium species’ Thermochemistries

In our recent publication, *Shirley et al.*[33], we have demonstrated the use of CompChem and RDF for investigating the thermodynamic properties of new titanium-oxygen molecules. In that paper, the python codes were implemented to make a SPARQL query to an early prototype of MolHub, i.e., “CoMo CompChem”. We successfully illustrated several advantages of the graph database. First, the relationships between chemical entities are clear and it is easy to define a graph pattern to match the desired criteria. Users with no specific training can quickly learn how to make a query and produce a useful result. Second, resources are uniquely labeled with a URL and exist online which make them promptly accessible from a small script to a large application. Third, visualization of the data is very useful as the molecule’s geometry reveals problems instantaneously if there are any. In MolHub an embedded Jmol applet is implemented allowing users to rapidly see the 3D structure of the molecules in the database and hence there is no need to use an external viewer.

**Figure 6:**
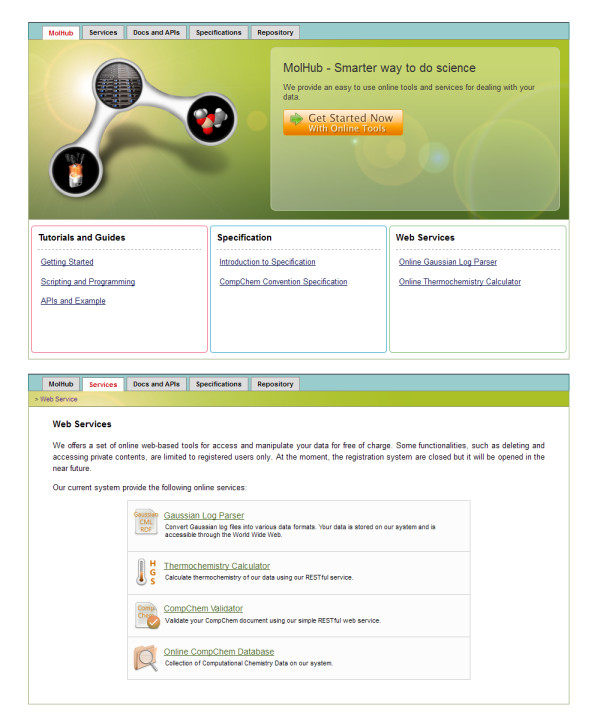
**A snippet of TiO**_**2**_ data in CompChem format consisting of two job modules.

In Figure 6, a snippet of a TiO_2_ molecule is shown. The calculations consist of two separate jobs, which are the geometry optimization and the frequency analysis. Our thermochemistry software, which runs on MolHub, reads the information in CompChem format and produces the thermodynamic properties, such as entropy (*S*), enthalpy (*H*), and specific heat capacity (_*Cp*_and _*Cv*_) and returns it as a downloadable web resource.

## Conclusions

An XML-based data storage format, CompChem, has been proposed to capture common aspects of computational chemistry modeling, i.e., model inputs (parameters), application model, calculation steps and model outputs (computed properties), into a well-formed structured manner. The new format minimizes the loss of information from its original source and adds semantics to the data set. The main contributions are: 

· The development of CompChem convention;

· The development of the validation tools, such as stylesheet and online CMLValidator;

· The digital repository, MolHub.

An important problem of the Semantic Web is that there is no generally-accepted standardized concept in use today, causing difficulty in the ontology design. This problem also applies to other chemistry domains. In order to insert a certain level of semantic information to CompChem, the concept of control vocabulary has been brought into use through a CML dictionary. The vocabulary terms used in CompChem can be documented and inserted to CompChem documents. The term modifiers, such as datatype, units, relationships, etc., can be added into a CML dictionary providing additional instructions to the processing software. The recent work by *Shirley et al.*[33] uses this method to process thermochemistry as part of an automated species screening investigation. However, we have yet to finalize a formal computational chemistry ontology. It is clear that the development of such an ontology cannot be undertaken by an individual, but must be driven by the community and experts in related fields in order to guarantee that it will be of benefit to the maximum number of people and therefore widely adopted.

For data validation, a rule-based schema language for CompChem has been developed to ensure that computational chemistry data is formed according to our specifications. The rule-based schema is developed using the XSLT standard and provided in the form of a stylesheet which can be processed separately from CML grammar-based validation using any XSLT processor. Although CompChem rules in the stylesheet can check for all the structural details, it cannot be used to check the validity of contents. For example, it cannot test whether the data type of a property for the associated term matches the data type defined in a dictionary. Such an assertion can be easily added to the stylesheet. A new method may be employed to solve this problem in future work.

## Availability and requirements

The CompChem Convention is available at http://www.xml-cml.org/convention/compchem and the CompChem dictionary is available at http://www.xml-cml.org/dictionary/compchem/. The code of CompChem validation stylesheet is available at https://bitbucket.org/wwmm/cml-specs and the CMLValidator is available at http://bitbucket.org/cml/cmllite-validator-code.

## Abbreviations

CML, Chemical Markup Language; CompChem, CML for computational chemistry; XML, eXtensible Markup Language; CMLXOM, A Java XML Object Model library for CML; Jumbo6, A set of chemistry libraries which provide abilities to manipulate CMLXOM; Jumbo-Converter, A set of libraries (“converters”) which provide conversion to and from CML; CMLValidator, A CML library for CML Convention validation; XSD, XML Schema Definition; DTD, Document Type Definition; W3C, The World Wide Web Consortium; CMLSpec, CML for spectral data; CMLReact, CML for chemical reactions; PML, Polymer Markup Language; QName, A Qualified Name as defined in the XML specifications; URL, Uniform Resource Locator; URI, Uniform Resource Identifier; SGML, Standard Generalized Markup Language; XPath, A syntax for defining parts of an XML document; XSL, eXtensible Stylesheet Language; XSLT, XSL Transformations; DCMI, Dublin Core©Metadata; OAI-ORE, Object Reuse and Exchange standards; NMR, Nuclear Magnetic Resonance; MolHub, An online infrastructure for chemical data (http://como.cheng.cam.ac.uk/ molhub/); RDF, Resource Description Framework; HTTP, Hypertext Transfer Protocol; REST, REpresentational State Transfer; API, Application Programming Interface; Ajax, Asynchronous JavaScript and XML; OWL, Web Ontology Language; N3, Notation3; PNG, Portable Network Graphics; SPARQL, SPARQL Protocol and RDF Query Language; OpenRDF, An RDF Schema-based Repository and Querying facility (http://www.openrdf.org/).

## Competing interests

The authors declare that they have no competing interests.

## Authors’ contributions

WP and JAT developed the specification for CompChem, created the codes and CompChem dictionary. WP wrote the manuscript and was checked by MK and PMR. All authors read and approved the final manuscript.

## References

[B1] Frisch MJ Trucks GW Schlegel HB Scuseria GE Robb MA Cheeseman JR Montgomery JAJr Vreven T Kudin KN Burant JC Millam JM Iyengar SS Tomasi J Barone V Mennucci B Cossi M Scalmani G Rega N Petersson GA Nakatsuji H Hada M Ehara M Toyota K Fukuda R Hasegawa J Ishida M Nakajima T Honda Y Kitao O Nakai H Klene M Li X Knox JE Hratchian HP Cross JB Bakken V Adamo C Jaramillo J Gomperts R Stratmann RE Yazyev O Austin AJ Cammi R Pomelli C Ochterski JW Ayala PY Morokuma K Voth GA Salvador P Dannenberg JJ Zakrzewski VG Dapprich S Daniels AD Strain MC Farkas O Malick DK Rabuck AD Raghavachari K Foresman JB Ortiz JV Cui Q Baboul AG Clifford S Cioslowski J Stefanov BB Liu G Liashenko A Piskorz P Komaromi I Martin RL Fox DJ Keith T Al-Laham MA Peng CY Nanayakkara A Challacombe M Gill PMW Johnson B Chen W Wong MW Gonzalez C Pople JA Gaussian 03, Revision C.02 2003 2004 Gaussian, Inc., Wallingford, CT

[B2] Schmidt M Baldridge K Boatz J Elbert S Gordon M Jensen J Koseki S Matsunaga N Nguyen K SSu T Windus DupuisM Montgomery J General Atomic and Molecular Electronic Structure System J Comput Chem 1993 14 1347 1363 10.1002/jcc.540141112

[B3] Guest MF Bush IJ Van Dam HJJ Sherwood P Thomas JMH Van Lenthe Havenith RWA Kendrick J The GAMESS-UK electronic structure package: algorithms, developments and applications Mol Phy 2005 103 6–8 719 747

[B4] Song J Building Robust Chemical Reaction Mechanisms: Next Generation of Automatic Model Construction Software PhD thesis 2004 Massachusetts Institute of, Technology, Cambridge, MA, USA http://hdl.handle.net/1721.1/30058

[B5] Wakelin J Murray-Rust P Tyrrell S Zhang Y Rzepa HS García A CML tools and information ow in atomic scale simulations Mol Simul 2005 31 5 315 322 10.1080/08927020500065850

[B6] Bray T Paoli J Sperberg-McQueen CM Maler E Yergeau F Extensible Markup Language (XML) 1.0 (Fifth Edition), W3C 2008 http://www.w3.org/TR/xml/

[B7] Murray-Rust P Rzepa HS Chemical Markup, XML , and the Worldwide Web. 1. Basic Principles J Chem Inf Comput Sci 1999 39 6 928 942 10.1021/ci990052b

[B8] Murray-Rust P Rzepa HS Chemical Markup, XML and theWorld-WideWeb. 2. Information Objects and the CMLDOM J Chem Inf Comput Sci 2001 41 5 1113 1123 10.1021/ci000404a 11604012

[B9] Gkoutos GV Murray-Rust P Rzepa HS Wright M Chemical Markup, XML , and the World-Wide Web. 3. Toward a Signed Semantic Chemical Web of Trust J Chem Inf Comput Sci 2001 41 5 1124 1130 10.1021/ci000406v 11604013

[B10] Murray-Rust P Rzepa HS Chemical Markup, XML , and the World Wide Web. 4. CML Schema J Chem Inf Comput Sci 2003 43 3 757 772 10.1021/ci0256541 12767134

[B11] Townsend J Murray-Rust P CMLLite: a design philosophy for CML J Cheminformatics 2011 3 39 10.1186/1758-2946-3-39 PMC3205043 21999395

[B12] Murray-Rust P Adams S Downing J Townsend J Zhang Y The semantic architecture of theWorld-Wide Molecular Matrix (WWMM) J Cheminformatics 2011 3 42 10.1186/1758-2946-3-42 PMC3205046 21999475

[B13] Murray-Rust P Townsend J Adams S Phadungsukanan W Thomas J The semantics of Chemical Markup Language (CML): dictionaries and conventions J Cheminformatics 2011 3 43 10.1186/1758-2946-3-43 PMC3206453 21999509

[B14] CMLXOM . [Online; accessed 20-December-2011]. [https://bitbucket.org/wwmm/cmlxom/]

[B15] Jumbo6 . [Online; accessed 20-December-2011]. [https://bitbucket.org/wwmm/jumbo6/]

[B16] JUMBO-Converters . [Online; accessed 20-December-2011]. [https://bitbucket.org/wwmm/jumbo-converters/]

[B17] CMLValidator service . [Online; accessed 20-December-2011]. [http://validator.xml-cml.org/]

[B18] O’Boyle N Banck M James C Morley C Vandermeersch T Hutchison G OpenBabel: An open chemical toolbox J Cheminformatics 2011 3 33 10.1186/1758-2946-3-33 PMC3198950 21982300

[B19] O’Boyle N Morley C Hutchison G Pybel: a Python wrapper for the OpenBabel cheminformatics toolkit Chem Cent J 2008 2 5 10.1186/1752-153X-2-5 18328109 PMC2270842

[B20] **Jmol: an open-source Java viewer for chemical structures in 3D**. [Online; accessed 24-October-2011]. [http://www.jmol.org/]

[B21] **Avogadro: an open-source molecular builder and visualization tool. Version 1.0.3**. [Online; accessed 25-April-2011]. [http://avogadro.openmolecules.net/]

[B22] Thompson HS Beech D Maloney M Mendelsohn N XML Schema Part 1: Structures Second Edition, W3C Recommendation 2004 [Online; accessed 21-December-2011]. [http://www.w3.org/TR/xmlschema-1/]

[B23] w3schools Introduction to XML Schema [Online; accessed 21-December-2011]. [http://www.w3schools.com/schema/schemaintro.asp]

[B24] Murray-Rust P Rzepa H Chemical Markup Language (CML) Schema version 3 [Online; accessed 24-December-2011]. [http://www.xml-cml.org/schema/] 10.1021/ci0256541 12767134

[B25] Holliday GL Murray-Rust P Rzepa HS Chemical Markup, XML , and the World Wide Web. 6. CML- React, an XML Vocabulary for Chemical Reactions J Chem Inf Model 2006 46 145 157 10.1021/ci0502698 16426051

[B26] Kuhn S Helmus T Lancashire RJ Murray-Rust P Rzepa HS Steinbeck C Willighagen EL Chemical, Markup, XML and the World Wide Web. 7. CMLSpect, an XML Vocabulary for Spectral Data J Chem Inf Model 2007 47 6 2015 2034 10.1021/ci600531a 17887743

[B27] Day N Downing J Adams S England NW Murray-Rust P CrystalEye [Online; accessed 26-December-2011]. [http://wwmm.ch.cam.ac.uk/crystaleye/]

[B28] Adams N Winter J Murray-Rust P Rzepa HS Chemical Markup, XML and the World-Wide Web. 8. Polymer Markup Language J Chem Inf Model 2008 48 11 2118 2128 10.1021/ci8002123 18991372

[B29] Bray T Hollander D Layman A Tobin R Thompson H S Rzepa CrystalEye 2009 [Online; accessed 26-December-2011]. [http://www.w3.org/TR/xml-names/]

[B30] Totton TS Shirley R Kraft M First-principles thermochemistry for the combustion of in a methane flame Proc Combust Inst 2011 33 493 500 10.1016/j.proci.2010.05.011

[B31] West RH Beran GJO Green WH Kraft M First-Principles Thermochemistry for the Production of TiO2 from TiCl4 J Phys Chem A 2007 111 18 3560 3565 10.1021/jp0661950 17441693

[B32] Shirley R Liu Y Totton TS West RH Kraft M First-Principles Thermochemistry for the Combustion of a TiCl4 and AlCl3 Mixture J Phys Chem A 2009 113 49 13790 13796 10.1021/jp905244w 19888740

[B33] Shirley R Phadungsukanan W Kraft M Downing J Day NE Murray-Rust P First-Principles Thermochem- istry for Gas Phase Species in an Industrial Rutile Chlorinator J Phys Chem A 2010 114 43 11825 11832 10.1021/jp106795p 20923209

[B34] Phadungsukanan W Shekar S Shirley R Sander M West RH Kraft M First-Principles Thermochemistry for Silicon Species in the Decomposition of Tetraethoxysilane J Phys Chem A 2009 113 31 9041 9049 10.1021/jp905494s 19603756

[B35] Berglund A Boag S Chamberlin D Fernández MF Kay M Robie J Siméon J XML Path Language (XPath) 2.0 (Second Edition) 2010 [Online; accessed 26-December-2011]. [http://www.w3.org/TR/xpath20/]

[B36] Kay M XSL Transformations (XSLT) Version 2.0 2007 [Online; accessed 26-December-2011]. [http://www.w3.org/TR/xslt20/]

[B37] Bradner S Key words for use in RFCs to Indicate Requirement Levels 1997 [Online; accessed 24-December-2011]. [http://www.ietf.org/rfc/rfc2119.txt]

[B38] Manola F Miller E Resource Description Framework (RDF) Primer 2004 [Online; accessed 6-February-2012]. [http://www.w3.org/TR/rdf-primer/]

[B39] **OWL 2 Web Ontology Language**, 2009. [Online; accessed 6-February-2012]. [http://www.w3.org/TR/owl2-overview/]

[B40] **OpenRDF - Aduna Software**. [Online; accessed 11-May-2012]. [http://www.openrdf.org/]

[B41] Prud’hommeaux E Seaborne A SPARQL Query Language for RDF 2008 [Online; accessed 11-May-2012].[http://www.w3.org/TR/rdf-sparql-query/]

